# Crystal structure of 4-(prop-2-yn­yloxy)-2,2,6,6-tetra­methyl­piperidin-1-ox­yl

**DOI:** 10.1107/S1600536814017991

**Published:** 2014-08-09

**Authors:** Shailesh K. Goswami, Lyall R. Hanton, C. John McAdam, Stephen C. Moratti, Jim Simpson

**Affiliations:** a Department of Chemistry, University of Otago, PO Box 56, Dunedin, New Zealand

**Keywords:** crystal structure, TEMPO derivative, C—H⋯alkyne contact

## Abstract

The title compound, C_12_H_20_NO_2_, was synthesized from 4-hy­droxy-2,2,6,6-tetra­methyl­piperidin-1-oxyl (hy­droxy-TEMPO) and propargyl bromide. The six-membered ring adopts a flattened chair conformation and carries a propyn­yloxy substituent in an equatorial orientation at the 4-position. The N—O bond length of the piperidin-1-oxyl unit is 1.289 (3) Å. In the crystal, C—H⋯O hydrogen bonds combine with unusual C—H⋯π inter­actions involving the alkyne unit as acceptor to generate a three-dimensional network.

## Abstract 2

The structure of a TEMPO derivative with a propyn­yloxy substituent at the 4-position of the piperidine ring is reported. The crystal packing features an unusual C—H⋯π inter­action involving the triple bond of the propyne group which combines with C—H⋯O hydrogen bonds to stack the mol­ecules along the *b*-axis direction.

## Chemical context   

TEMPO, 2,2,6,6-tetra­methyl­piperidin-1-oxyl, and its derivatives have attracted significant inter­est in recent years as functional organic radicals with considerable chemical stability (Soegiarto *et al.*, 2011 ▶). They are known to exhibit both ferromagnetism and anti­ferromagnetism at low temperatures (Togashi *et al.*, 1996 ▶; Ishida *et al.*, 1995 ▶), and the effect of inter­molecular contacts on their magnetic properties has been examined (Iwasaki *et al.*, 1999*a* ▶,*b* ▶). TEMPO and its derivatives have been utilized in applications as diverse as catalysis in organic synthesis (Zhao *et al.*, 2005 ▶), pulsed electron–electron double-resonance (PELDOR) spectroscopy (Bode *et al.*, 2007 ▶), and use as qubits (quantum bits) in quantum computing (Nakazawa *et al.*, 2012 ▶).
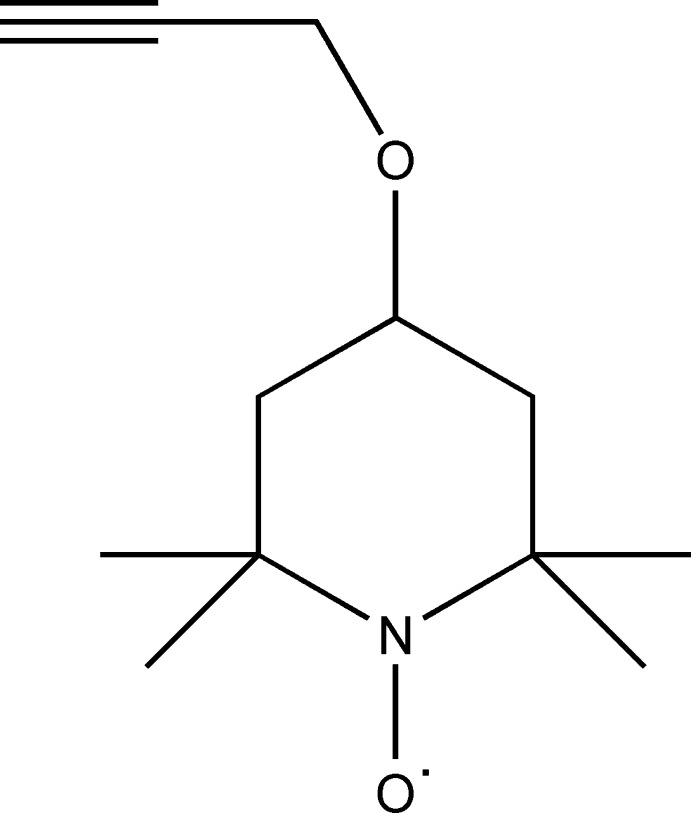


Our inter­est in TEMPO derivatives is as reversible redox-active subunits in polymer-gel actuators (Goswami *et al.*, 2013 ▶). In particular, the alkyne group present in the title compound, (1), allows us to utilize the versatile CuAAC ‘click’ cyclo­addition with organic azides (Hein & Fokin, 2010 ▶; Lewis *et al.*, 2013 ▶) as a means to attach the TEMPO unit to the gel skeleton.

## Structural commentary   

**Figure 1:**
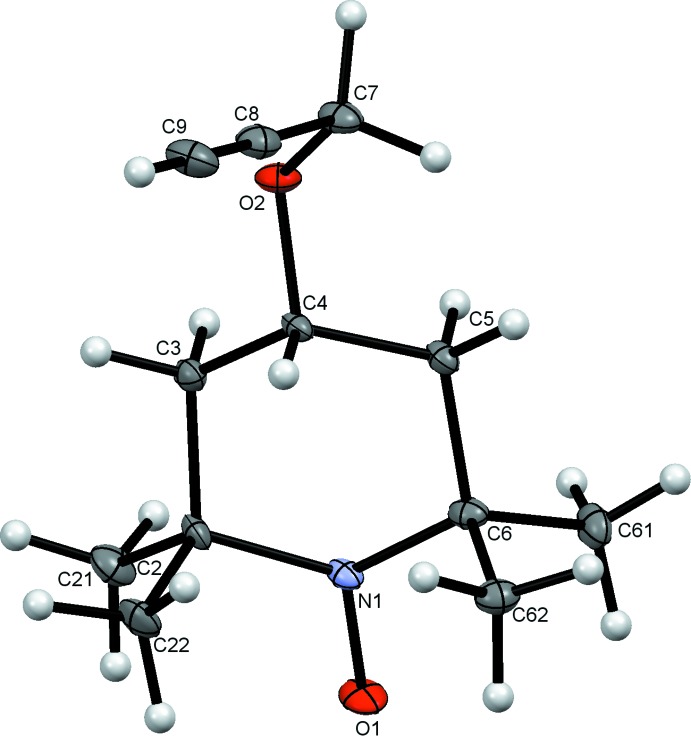
The structure of (1), showing the atom numbering and with displacement ellipsoids drawn at the 50% probability level.

The structure of (1) and its atom numbering are shown in Fig. 1 ▶. The mol­ecule comprises a standard TEMPO unit with a propyn­yloxy substituent at the 4-position. The N1/C2–C6 ring adopts a flattened chair conformation with the C4 atom 0.706 (4) Å from the best fit plane through the remaining four C atoms, while N1 lies only 0.384 (4) Å from the plane in the opposite direction. The propynyl C7–C9 unit points away from this plane in the same direction as C4, with C7—C8—C9 = 178.6 (3)°. The N—O bond is 1.289 (3) Å long, which compares favorably with the average value of 1.285 (18) Å for other TEMPO structures (Macrae *et al.*, 2008 ▶).

## Supra­molecular features   

**Figure 2:**
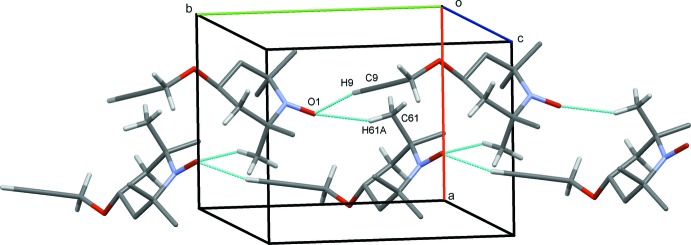
Double chains formed from mol­ecules of (1) along *b*. In this and subsequent Figures, C—H⋯O hydrogen bonds are drawn as dashed lines and H atoms bound to atoms not involved in hydrogen bonding are not shown.

**Figure 3:**
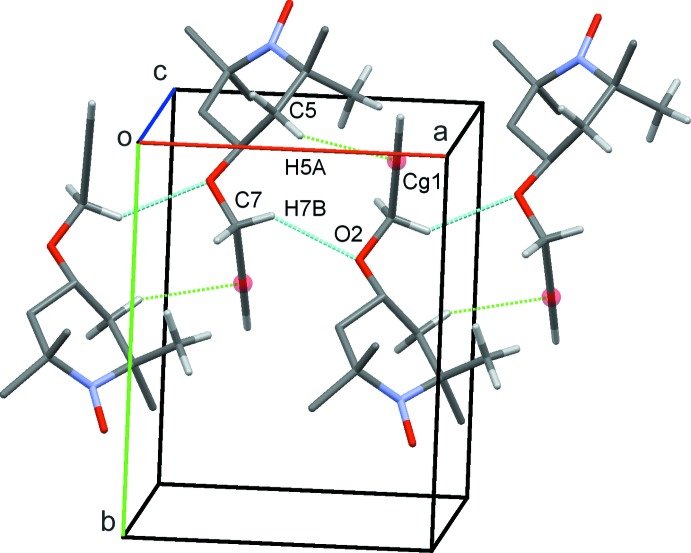
Zigzag chains formed along *a* from C—H⋯O and C—H⋯π (green dotted lines) contacts. The mid-point of the C8=C9 triple bond is shown as a red sphere.

**Figure 4:**
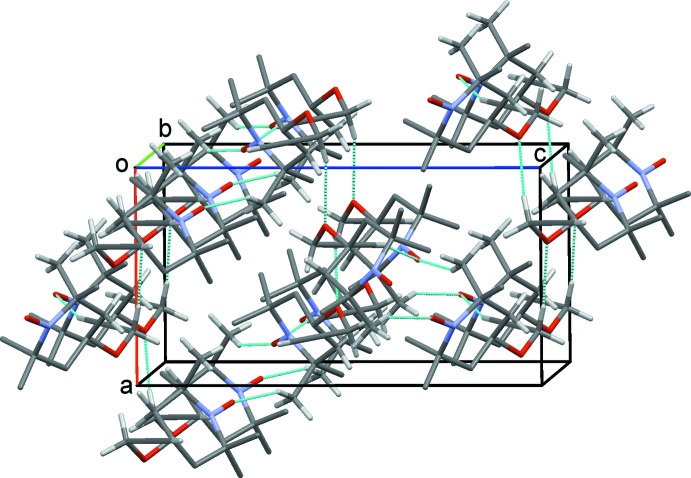
The overall packing for (1), viewed along the *b* axis.

In the crystal structure of (1), C9—H9⋯O1 hydrogen bonds link mol­ecules into *C*(9) chains along *b* (Table 1 ▶). Additional C61—H61*A*⋯O1 contacts form 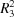
(16) rings, resulting in double chains of mol­ecules along *b* (Fig. 2 ▶). In an almost orthogonal direction, C7—H7*B*⋯O2 hydrogen bonds form *C*(3) chains along *a*. An inter­esting feature of these latter contacts is the support provided by C5—H5*B*⋯*Cg* inter­actions (*Cg* is the mid-point of the C8—C9 bond) involving the alkyne unit (Fig. 3 ▶). Such contacts are often overlooked, but they have been reported previously for both terminal and non-terminal alkyne systems (Banerjee *et al.*, 2006 ▶; Thakur *et al.*, 2010 ▶; McAdam *et al.*, 2012 ▶). Overall, these contacts generate a three-dimensional network with mol­ecules stacked in inter­connected columns along the *b* axis (Fig. 4 ▶).

## Database survey   

The Cambridge Structural Database (CSD; Version 5.35, November 2013 with 2 updates; Allen, 2002 ▶) reveals a total of 175 structures of TEMPO and its derivatives. However, structures of alk­oxy-TEMPO derivatives are rare with only a single example, albeit in two separate papers in which Polovyanenko *et al.* (2008 ▶) and Soegiarto *et al.* (2011 ▶) report the structure of 4-(meth­oxy)-TEMPO, 4-(meth­oxy)-2,2,6,6-tetra­methyl­piperidin-1-oxyl. The first paper examines the TEMPO derivative as an inclusion complex of *p*-hexa­noyl calix[4]arene (C_6_OH), and investigates the magnetism and orientation dependent motion of the encapsulated radical. In the second, the mol­ecule is included in the cavities of two porous frameworks derived from guanidinium cations and two organodi­sulfonate anions; the magnetic behaviour of the radical guest is investigated. Arylo­yloxy-TEMPO derivatives are more abundant with 19 entries in the CSD (see, for example, Pang *et al.*, 2013 ▶; Nakazawa *et al.*, 2012 ▶; Akutsu *et al.*, 2005 ▶). Again, the focus is very much on the magnetic properties of the materials.

## Synthesis and crystallization   

Synthesis and characterization (IR and mass spectroscopy) are as previously described (Gheorghe *et al.*, 2006 ▶; Kulis *et al.*, 2009 ▶). Colourless blocks were obtained from diethyl ether solution at room temperature. Analysis calculated for C_12_H_20_NO_2_: C 68.54, H 9.59, N 6.66%; found: C 68.57, H 9.66, N 6.68%.

## Refinement   

Crystal data, data collection and structure refinement details are summarized in Table 2 ▶. With no heavy atom in the non-centrosymmetric structure, the absolute structure could not be reliably determined. Friedel opposites were not, however, merged. All H atoms were refined using a riding model, with C—H = 0.99 Å and *U*_iso_(H) = 1.2*U*_eq_(C) for methyl­ene H atoms, C—H = 1.00 Å and *U*_iso_(H) = 1.2*U*_eq_(C) for methine H atoms, C—H = 0.98 Å and *U*_iso_(H) = 1.5*U*_eq_(C) for methyl H atoms, and C—H = 0.95 Å and *U*_iso_(H) = 1.2*U*_eq_(C) for the terminal alkyne H atom. Anisiotropic refinement of the non-H atoms was constrained using the ISOR command in *SHELXL* to prevent atoms becoming non-positive definite. 10 reflections with *F*_o_ >> *F*_c_ were omitted from the final refinement cycles.

## Supplementary Material

Crystal structure: contains datablock(s) global, 1. DOI: 10.1107/S1600536814017991/hb7267sup1.cif

Structure factors: contains datablock(s) 1. DOI: 10.1107/S1600536814017991/hb72671sup2.hkl

CCDC reference: 1017949

Additional supporting information:  crystallographic information; 3D view; checkCIF report

## Figures and Tables

**Table 1:** Hydrogen-bond geometry (Å, °) *Cg* is the mid-point of the C8–C9 bond.

*D*—H⋯*A*	*D*—H	H⋯*A*	*D*⋯*A*	*D*—H⋯*A*
C9—H9⋯O1^i^	0.95	2.28	3.205 (4)	163
C7—H7*B*⋯O2^ii^	0.99	2.52	3.298 (4)	135
C61—H61*A*⋯O1^iii^	0.98	2.56	3.481 (4)	157
C5—H5*B*⋯*Cg* ^iv^	0.99	2.93	3.885 (4)	156

Symmetry codes: (i) 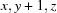; (ii) 
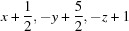
; (iii) 
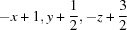
; (iv) 
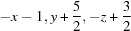
.

**Table 2:** Experimental details

Crystal data	
Chemical formula	C_12_H_20_NO_2_
*M*_r_	210.29
Crystal system, space group	Orthorhombic, *P*2_1_2_1_2_1_
Temperature (K)	100
*a*, *b*, *c* (Å)	7.94506 (13), 10.17919 (16), 14.8052 (3)
*V* (Å^3^)	1197.36 (4)
*Z*	4
Radiation type	Cu *K*α
μ (mm^−1^)	0.63
Crystal size (mm)	0.18 × 0.15 × 0.08

Data collection	
Diffractometer	Agilent SuperNova (Dual, Cu at zero, Atlas)
Absorption correction	Multi-scan (*CrysAlis PRO*; Agilent, 2013 ▶)
*T*_min_, *T*_max_	0.522, 1.000
No. of measured, independent and observed [*I* > 2σ(*I*)] reflections	6622, 2307, 2203
*R*_int_	0.046
(sin θ/λ)_max_ (Å^−1^)	0.624

Refinement	
*R*[*F* ^2^ > 2σ(*F* ^2^)], *wR*(*F* ^2^), *S*	0.043, 0.123, 1.15
No. of reflections	2307
No. of parameters	140
No. of restraints	90
H-atom treatment	H-atom parameters constrained
Δρ_max_, Δρ_min_ (e Å^−3^)	0.22, −0.28
Absolute structure	Flack *x* determined using 858 quotients [(*I* ^+^)−(*I* ^−^)]/[(*I* ^+^)+(*I* ^−^)] (Parsons & Flack, 2004 ▶)
Absolute structure parameter	0.0 (3)

Computer programs: *CrysAlis PRO* (Agilent, 2013 ▶), *SIR2011* (Burla *et al.*, 2012 ▶), *SHELXL2013* (Sheldrick, 2008 ▶), *TITAN2000* (Hunter & Simpson, 1999 ▶), *Mercury* (Macrae *et al.*, 2008 ▶), *enCIFer* (Allen *et al.*, 2004 ▶), *PLATON* (Spek, 2009 ▶) and *publCIF* (Westrip 2010 ▶).

## supplementary crystallographic information

### Crystal data

**Table:**

C_12_H_20_NO_2_	*D*_x_ = 1.167 Mg m^−^^3^
*M**_r_* = 210.29	Cu *K*α radiation, λ = 1.54184 Å
Orthorhombic, *P*2_1_2_1_2_1_	Cell parameters from 4862 reflections
*a* = 7.94506 (13) Å	θ = 5.3–74.2°
*b* = 10.17919 (16) Å	µ = 0.63 mm^−^^1^
*c* = 14.8052 (3) Å	*T* = 100 K
*V* = 1197.36 (4) Å^3^	Block, colourless
*Z* = 4	0.18 × 0.15 × 0.08 mm
*F*(000) = 460	

### Data collection

**Table:**

Agilent SuperNova (Dual, Cu at zero, Atlas) diffractometer	2307 independent reflections
Radiation source: SuperNova (Cu) X-ray Source	2203 reflections with *I* > 2σ(*I*)
Mirror monochromator	*R*_int_ = 0.046
Detector resolution: 5.1725 pixels mm^-1^	θ_max_ = 74.3°, θ_min_ = 5.3°
ω scans	*h* = −9→9
Absorption correction: multi-scan (*CrysAlis PRO*; Agilent, 2013)	*k* = −12→12
*T*_min_ = 0.522, *T*_max_ = 1.000	*l* = −18→13
6622 measured reflections	

### Refinement

**Table:**

Refinement on *F*^2^	Hydrogen site location: inferred from neighbouring sites
Least-squares matrix: full	H-atom parameters constrained
*R*[*F*^2^ > 2σ(*F*^2^)] = 0.043	*w* = 1/[σ^2^(*F*_o_^2^) + (0.0376*P*)^2^ + 1.029*P*] where *P* = (*F*_o_^2^ + 2*F*_c_^2^)/3
*wR*(*F*^2^) = 0.123	(Δ/σ)_max_ < 0.001
*S* = 1.15	Δρ_max_ = 0.22 e Å^−^^3^
2307 reflections	Δρ_min_ = −0.28 e Å^−^^3^
140 parameters	Absolute structure: Flack *x* determined using 858 quotients [(*I*^+^)-(*I*^-^)]/[(*I*^+^)+(*I*^-^)] (Parsons & Flack, 2004)
90 restraints	Absolute structure parameter: 0.0 (3)

### Special details

**Table:**

Geometry. All e.s.d.'s (except the e.s.d. in the dihedral angle between two l.s. planes) are estimated using the full covariance matrix. The cell e.s.d.'s are taken into account individually in the estimation of e.s.d.'s in distances, angles and torsion angles; correlations between e.s.d.'s in cell parameters are only used when they are defined by crystal symmetry. An approximate (isotropic) treatment of cell e.s.d.'s is used for estimating e.s.d.'s involving l.s. planes.

### Fractional atomic coordinates and isotropic or equivalent isotropic displacement parameters (Å^2^)

**Table:**

	*x*	*y*	*z*	*U*_iso_*/*U*_eq_	
O1	0.3936 (3)	0.73046 (19)	0.71556 (15)	0.0174 (5)	
N1	0.3444 (3)	0.8315 (2)	0.67027 (16)	0.0107 (5)	
C2	0.2036 (4)	0.9093 (3)	0.71116 (19)	0.0116 (6)	
C21	0.2698 (4)	0.9827 (3)	0.7945 (2)	0.0169 (6)	
H21A	0.3310	0.9214	0.8335	0.025*	
H21B	0.1750	1.0205	0.8280	0.025*	
H21C	0.3457	1.0533	0.7753	0.025*	
C22	0.0670 (4)	0.8118 (3)	0.7398 (2)	0.0179 (7)	
H22A	0.0301	0.7611	0.6872	0.027*	
H22B	−0.0289	0.8599	0.7651	0.027*	
H22C	0.1125	0.7520	0.7856	0.027*	
C3	0.1325 (3)	1.0046 (3)	0.6408 (2)	0.0119 (6)	
H3A	0.0654	0.9543	0.5964	0.014*	
H3B	0.0558	1.0671	0.6714	0.014*	
C4	0.2663 (4)	1.0812 (3)	0.59126 (19)	0.0099 (6)	
H4	0.3331	1.1364	0.6341	0.012*	
C5	0.3796 (4)	0.9843 (3)	0.54137 (19)	0.0112 (6)	
H5A	0.4664	1.0342	0.5078	0.013*	
H5B	0.3110	0.9357	0.4966	0.013*	
C6	0.4674 (4)	0.8850 (3)	0.6036 (2)	0.0114 (6)	
C61	0.6167 (4)	0.9461 (3)	0.6539 (2)	0.0152 (6)	
H61A	0.5803	1.0270	0.6840	0.023*	
H61B	0.7065	0.9665	0.6108	0.023*	
H61C	0.6587	0.8839	0.6991	0.023*	
C62	0.5300 (4)	0.7698 (3)	0.5464 (2)	0.0168 (6)	
H62A	0.5934	0.7087	0.5846	0.025*	
H62B	0.6031	0.8028	0.4982	0.025*	
H62C	0.4335	0.7240	0.5197	0.025*	
O2	0.1772 (3)	1.16202 (19)	0.52724 (14)	0.0140 (5)	
C7	0.2733 (4)	1.2682 (3)	0.4916 (2)	0.0149 (6)	
H7A	0.2201	1.2992	0.4350	0.018*	
H7B	0.3877	1.2362	0.4765	0.018*	
C8	0.2872 (4)	1.3793 (3)	0.5551 (2)	0.0160 (6)	
C9	0.2975 (4)	1.4705 (3)	0.6048 (2)	0.0198 (7)	
H9	0.3058	1.5435	0.6445	0.024*	

### Atomic displacement parameters (Å^2^)

**Table:**

	*U*^11^	*U*^22^	*U*^33^	*U*^12^	*U*^13^	*U*^23^
O1	0.0281 (11)	0.0083 (9)	0.0159 (11)	0.0025 (9)	0.0015 (10)	0.0060 (8)
N1	0.0172 (11)	0.0066 (10)	0.0085 (10)	0.0001 (9)	0.0007 (10)	0.0011 (8)
C2	0.0163 (13)	0.0110 (12)	0.0075 (12)	−0.0005 (11)	0.0037 (12)	0.0002 (10)
C21	0.0245 (15)	0.0169 (14)	0.0092 (14)	−0.0002 (12)	−0.0002 (13)	−0.0026 (11)
C22	0.0208 (15)	0.0190 (15)	0.0140 (15)	−0.0056 (12)	0.0028 (13)	0.0044 (12)
C3	0.0145 (12)	0.0110 (12)	0.0101 (13)	0.0020 (10)	0.0011 (11)	−0.0008 (10)
C4	0.0148 (13)	0.0087 (12)	0.0061 (12)	0.0005 (10)	0.0000 (11)	0.0012 (9)
C5	0.0160 (12)	0.0092 (12)	0.0084 (13)	−0.0001 (10)	0.0009 (11)	0.0009 (10)
C6	0.0161 (12)	0.0090 (12)	0.0090 (13)	0.0008 (10)	−0.0008 (11)	0.0015 (10)
C61	0.0165 (13)	0.0132 (13)	0.0160 (15)	−0.0004 (11)	−0.0003 (13)	0.0025 (11)
C62	0.0229 (14)	0.0122 (13)	0.0153 (16)	0.0036 (11)	0.0048 (13)	−0.0019 (11)
O2	0.0178 (10)	0.0102 (9)	0.0140 (10)	0.0000 (8)	−0.0032 (9)	0.0041 (7)
C7	0.0209 (14)	0.0105 (12)	0.0133 (14)	0.0004 (11)	−0.0017 (12)	0.0040 (10)
C8	0.0178 (13)	0.0139 (13)	0.0161 (14)	−0.0007 (11)	−0.0008 (12)	0.0057 (11)
C9	0.0275 (15)	0.0155 (15)	0.0164 (15)	−0.0025 (12)	−0.0026 (14)	0.0022 (12)

### Geometric parameters (Å, º)

**Table:**

O1—N1	1.289 (3)	C5—C6	1.535 (4)
N1—C6	1.492 (4)	C5—H5A	0.9900
N1—C2	1.498 (4)	C5—H5B	0.9900
C2—C3	1.531 (4)	C6—C62	1.530 (4)
C2—C22	1.531 (4)	C6—C61	1.532 (4)
C2—C21	1.536 (4)	C61—H61A	0.9800
C21—H21A	0.9800	C61—H61B	0.9800
C21—H21B	0.9800	C61—H61C	0.9800
C21—H21C	0.9800	C62—H62A	0.9800
C22—H22A	0.9800	C62—H62B	0.9800
C22—H22B	0.9800	C62—H62C	0.9800
C22—H22C	0.9800	O2—C7	1.424 (3)
C3—C4	1.509 (4)	C7—C8	1.475 (4)
C3—H3A	0.9900	C7—H7A	0.9900
C3—H3B	0.9900	C7—H7B	0.9900
C4—O2	1.441 (3)	C8—C9	1.187 (5)
C4—C5	1.526 (4)	C9—H9	0.9500
C4—H4	1.0000		
			
O1—N1—C6	115.9 (2)	C4—C5—C6	113.8 (2)
O1—N1—C2	116.0 (2)	C4—C5—H5A	108.8
C6—N1—C2	124.3 (2)	C6—C5—H5A	108.8
N1—C2—C3	109.6 (2)	C4—C5—H5B	108.8
N1—C2—C22	107.3 (2)	C6—C5—H5B	108.8
C3—C2—C22	109.7 (2)	H5A—C5—H5B	107.7
N1—C2—C21	109.1 (2)	N1—C6—C62	107.4 (2)
C3—C2—C21	111.4 (2)	N1—C6—C61	109.5 (2)
C22—C2—C21	109.6 (2)	C62—C6—C61	109.2 (2)
C2—C21—H21A	109.5	N1—C6—C5	109.9 (2)
C2—C21—H21B	109.5	C62—C6—C5	108.7 (2)
H21A—C21—H21B	109.5	C61—C6—C5	112.1 (2)
C2—C21—H21C	109.5	C6—C61—H61A	109.5
H21A—C21—H21C	109.5	C6—C61—H61B	109.5
H21B—C21—H21C	109.5	H61A—C61—H61B	109.5
C2—C22—H22A	109.5	C6—C61—H61C	109.5
C2—C22—H22B	109.5	H61A—C61—H61C	109.5
H22A—C22—H22B	109.5	H61B—C61—H61C	109.5
C2—C22—H22C	109.5	C6—C62—H62A	109.5
H22A—C22—H22C	109.5	C6—C62—H62B	109.5
H22B—C22—H22C	109.5	H62A—C62—H62B	109.5
C4—C3—C2	113.5 (2)	C6—C62—H62C	109.5
C4—C3—H3A	108.9	H62A—C62—H62C	109.5
C2—C3—H3A	108.9	H62B—C62—H62C	109.5
C4—C3—H3B	108.9	C7—O2—C4	114.4 (2)
C2—C3—H3B	108.9	O2—C7—C8	112.7 (2)
H3A—C3—H3B	107.7	O2—C7—H7A	109.1
O2—C4—C3	105.6 (2)	C8—C7—H7A	109.1
O2—C4—C5	109.9 (2)	O2—C7—H7B	109.1
C3—C4—C5	108.5 (2)	C8—C7—H7B	109.1
O2—C4—H4	110.9	H7A—C7—H7B	107.8
C3—C4—H4	110.9	C9—C8—C7	178.6 (3)
C5—C4—H4	110.9	C8—C9—H9	180.0
			
O1—N1—C2—C3	−166.8 (2)	O1—N1—C6—C62	49.9 (3)
C6—N1—C2—C3	36.2 (4)	C2—N1—C6—C62	−153.1 (3)
O1—N1—C2—C22	−47.7 (3)	O1—N1—C6—C61	−68.6 (3)
C6—N1—C2—C22	155.3 (3)	C2—N1—C6—C61	88.4 (3)
O1—N1—C2—C21	71.0 (3)	O1—N1—C6—C5	167.9 (2)
C6—N1—C2—C21	−86.0 (3)	C2—N1—C6—C5	−35.1 (4)
N1—C2—C3—C4	−47.6 (3)	C4—C5—C6—N1	45.1 (3)
C22—C2—C3—C4	−165.2 (2)	C4—C5—C6—C62	162.3 (2)
C21—C2—C3—C4	73.2 (3)	C4—C5—C6—C61	−76.9 (3)
C2—C3—C4—O2	178.6 (2)	C3—C4—O2—C7	163.0 (2)
C2—C3—C4—C5	60.8 (3)	C5—C4—O2—C7	−80.2 (3)
O2—C4—C5—C6	−174.6 (2)	C4—O2—C7—C8	−77.8 (3)
C3—C4—C5—C6	−59.5 (3)		

### Hydrogen-bond geometry (Å, º)

Cg is the mid-point of the C8–C9 bond.

**Table:**

*D*—H···*A*	*D*—H	H···*A*	*D*···*A*	*D*—H···*A*
C9—H9···O1^i^	0.95	2.28	3.205 (4)	163
C7—H7*B*···O2^ii^	0.99	2.52	3.298 (4)	135
C61—H61*A*···O1^iii^	0.98	2.56	3.481 (4)	157
C5—H5*B*···*Cg*^iv^	0.99	2.93	3.885 (4)	156

Symmetry codes: (i) *x*, *y*+1, *z*; (ii) *x*+1/2, −*y*+5/2, −*z*+1; (iii) −*x*+1, *y*+1/2, −*z*+3/2; (iv) −*x*−1, *y*+5/2, −*z*+3/2.

## References

[bb1] Agilent (2013). *CrysAlis PRO* Agilent Technologies, Yarnton, England.

[bb2] Akutsu, H., Masaki, K., Mori, K., Yamada, J. & Nakatsuji, S. (2005). *Polyhedron*, **24**, 2126–2132.

[bb3] Allen, F. H. (2002). *Acta Cryst.* B**58**, 380–388. 10.1107/s0108768102003890 12037359

[bb4] Allen, F. H., Johnson, O., Shields, G. P., Smith, B. R. & Towler, M. (2004). *J. Appl. Cryst.* **37**, 335–338.

[bb5] Banerjee, R., Mondal, R., Howard, J. A. K. & Desiraju, G. R. (2006). *Cryst. Growth Des.* **6**, 999–1009.

[bb6] Bode, B. E., Margraf, D., Plackmeyer, J., Dumer, G., Prisner, T. F. & Schiemann, O. (2007). *J. Am. Chem. Soc.* **129**, 6736–6745. 10.1021/ja065787t 17487970

[bb7] Camalli, M., Carrozzini, B., Cascarano, G. L. & Giacovazzo, C. (2012). *J. Appl. Cryst.* **45**, 351–356.

[bb8] Gheorghe, A., Matsuno, A. & Reiser, O. (2006). *Adv. Synth. Catal.* **348**, 1016—1020.

[bb9] Goswami, S. K., McAdam, C. J., Lee, A. M. M., Hanton, L. R. & Moratti, S. C. (2013). *J. Mater. Chem. A*, **1**, 3415–3420.

[bb10] Hein, J. E. & Fokin, V. V. (2010). *Chem. Soc. Rev.* **39**, 1302—1315. 10.1039/b904091a PMC3073167 20309487

[bb11] Hunter, K. A. & Simpson, J. (1999). *TITAN2000* University of Otago, New Zealand.

[bb12] Ishida, T., Tomioka, K., Nogami, T., Yoshikawa, H., Yasui, M., Iwasaki, F., Takeda, N. & Ishikawa, M. (1995). *Chem. Phys. Lett.* **247**, 7–12.

[bb13] Iwasaki, F., Yoshikawa, J. H., Yamamoto, H., Kan-nari, E., Takada, K., Yasui, M., Ishida, T. & Nogami, T. (1999*a*). *Acta Cryst.* B**55**, 231–245. 10.1107/s0108768198012786 10927362

[bb14] Iwasaki, F., Yoshikawa, J. H., Yamamoto, H., Takada, K., Kan-nari, E., Yasui, M., Ishida, T. & Nogami, T. (1999*b*). *Acta Cryst.* B**55**, 1057–1067. 10.1107/s0108768199007326 10927447

[bb15] Kulis, J., Bell, C. A., Micallef, A. S., Jia, Z. & Monteiro, M. J. (2009). *Macromolecules*, **42**, 8218–8227.

[bb16] Lewis, J. E. M., McAdam, C. J., Gardiner, M. G. & Crowley, J. D. (2013). *Chem. Commun.* **49**, 3398–3400. 10.1039/c3cc41209a 23515345

[bb17] Macrae, C. F., Bruno, I. J., Chisholm, J. A., Edgington, P. R., McCabe, P., Pidcock, E., Rodriguez-Monge, L., Taylor, R., van de Streek, J. & Wood, P. A. (2008). *J. Appl. Cryst.* **41**, 466–470.

[bb18] McAdam, C. J., Cameron, S. A., Hanton, L. R., Manning, A. R., Moratti, S. C. & Simpson, J. (2012). *CrystEngComm*, **14**, 4369–4383.

[bb19] Nakazawa, S., Nishida, S., Ise, T., Yoshino, T., Mori, N., Rahimi, R. D., Sato, K., Morita, Y., Toyota, K., Shiomi, D., Kitagawa, M., Hara, H., Carl, P., Hofer, P. & Takui, T. (2012). *Angew. Chem. Int. Ed.* **51**, 9860–9864. 10.1002/anie.201204489 22936609

[bb20] Pang, X., Wang, H., Zhao, X. R. & Wei Jin, W. J. (2013). *Dalton Trans.*, **42**, 8788–8795. 10.1039/c3dt50191d 23640048

[bb21] Parsons, S. & Flack, H. (2004). *Acta Cryst.* A**60**, s61.

[bb22] Polovyanenko, D. N., Bagryanskaya, E. G., Schnegg, A., Mobius, K., Coleman, A. W., Ananchenko, G. S., Udachin, K. A. & Ripmeester, J. A. (2008). *Phys. Chem. Chem. Phys.* **10**, 5299–5307. 10.1039/b803296c 18728872

[bb23] Sheldrick, G. M. (2008). *Acta Cryst.* A**64**, 112–122. 10.1107/S0108767307043930 18156677

[bb24] Soegiarto, A. C., Yan, W., Kent, A. D. & Ward, M. D. (2011). *J. Mater. Chem.* **21**, 2204–2219.

[bb25] Spek, A. L. (2009). *Acta Cryst.* D**65**, 148–155. 10.1107/S090744490804362X PMC2631630 19171970

[bb26] Thakur, A., Adarsh, N. A., Chakraborty, A., Devi, M. & Ghosh, S. (2010). *J. Organomet. Chem.* **695**, 1059–1064.

[bb27] Togashi, K., Imachi, R., Tomioka, K., Tsuboi, H., Ishida, T., Nogami, T., Takeda, N. & Ishikawa, M. (1996). *Bull. Chem. Soc. Jpn*, **69**, 2821–2830.

[bb28] Westrip, S. P. (2010). *J. Appl. Cryst.* **43**, 920–925.

[bb29] Zhao, M. M., Li, J., Mano, E., Song, Z. J. & Tschaen, D. M. (2005). *Org. Synth.* **81**, 195–203.

